# Gamified Simulation for Onboarding Health Care Teams in Emergency Care: Development and Preliminary Feasibility Study

**DOI:** 10.2196/72202

**Published:** 2025-12-15

**Authors:** Stephane Gobron, Antoine Lestrade, Artan Sadiku, Alexandre Bentvelzen, Leila Ait Kaci, Jean-Michel Carrier, Emmanuelle Guyot, Pierre-Nicolas Carron

**Affiliations:** 1 Department of Computer Science, HE-Arc, School of Engineering, HES-SO University of Applied Sciences and Arts Western Switzerland, Espace de l'Europe 11, Neuchatel, 2000, Switzerland, +41 32 930 11 11; 2 Emergency Department, Lausanne University Hospital (CHUV), Lausanne, Switzerland; 3 Digital Kingdom, Vevey, Switzerland; 4 Medical Education Unit, School of Medicine, University of Lausanne, Lausanne, Switzerland; 5 Faculty of Medicine, University of Lausanne, , Lausanne, Switzerland

**Keywords:** personnel turnover, digital learning environment, feasibility studies, user acceptability, staff development, self-directed training, health technology implementation, simulation training, mobile phone

## Abstract

**Background:**

High staff turnover is a widespread issue across nearly all hospital departments, often exceeding 20% annually. This constant flux disrupts continuity of care and creates a recurring challenge: how to rapidly integrate new employees into complex clinical environments, both physically and functionally. Traditional onboarding methods struggle to meet this demand, particularly in services operating 24/7, such as emergency departments (EDs).

**Objective:**

This formative study presents the design and implementation of a web-based 3D gamified simulation platform aimed at improving staff onboarding in clinical environments. The paper outlines both the technical architecture—with guidance for hospital IT departments—and the acceptability and usability for permanent staff, who play a key role in ensuring onboarding continuity. We sought to assess whether such a tool could be autonomously managed and well received by health care professionals.

**Methods:**

The intervention consisted of 2 linked components: a real-time, browser-based 3D simulation replicating the hospital’s ED and a web-based quest editor allowing nontechnical staff to update training content. The system supports self-paced onboarding through location-based tasks, object searches, quizzes, and simulated staff interactions. Two preliminary usability studies were conducted: one with 37 ED staff members testing the 3D simulation and another with 9 users exploring the quest editor. Feedback was gathered through anonymous questionnaires and a descriptive analysis.

**Results:**

Early results showed high feasibility and acceptability. Among 3D simulation testers (n=37), 90% (33/37) found the tool helpful for understanding the department’s structure, and 81% (30/37) believed it would be useful for new staff. The inclusion of personal anecdotes and gamified tasks was viewed as engaging and motivating. The quest editor (n=9) was positively rated by 91% (8/9) of users, who appreciated the ability to autonomously update content without IT support. These findings support the dual promise of the platform (ie, pedagogical flexibility and technical sustainability).

**Conclusions:**

This work demonstrates the feasibility of a gamified simulation platform designed for high-turnover clinical environments. It highlights both the operational deployment framework and the early acceptability among key staff members. While further validation with actual new hires is needed, this formative study shows promising potential for generalization beyond emergency care. The modular and editable nature of the system makes it a viable solution for scalable onboarding in other hospital departments.

## Introduction

### Context and Relevant Issues

High staff turnover is a persistent and well-documented issue in hospital settings, particularly in emergency departments (EDs), where annual turnover rates often exceed 20% [1,2]. This situation gives rise to several major challenges: reduced team cohesion, loss of institutional knowledge, increased demands on permanent staff to repeatedly onboard newcomers, and the rapid familiarization of newcomers with the premises, equipment, and colleagues. Despite the prevalence of this issue, existing onboarding strategies struggle to provide scalable, flexible, and engaging solutions, especially in services operating 24/7 with complex spatial and organizational structures.

### Toward a New Paradigm in Onboarding

**Figure 1.**
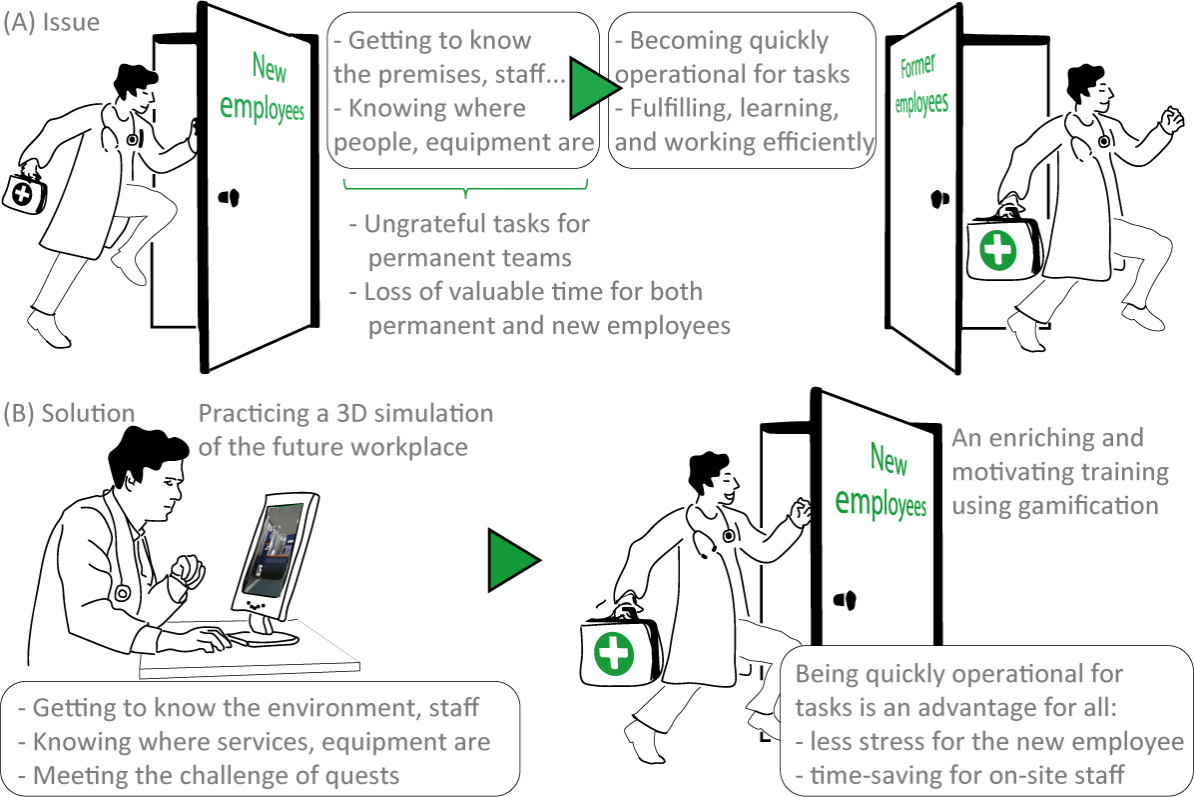
Challenges in integrating new employees in the emergency department (ED). (A) This figure summarizes the essence of the problem: similar to any workplace, new employees arrive at the ED and spend a certain amount of time getting to know the place, the equipment, and their colleagues before becoming operational. The difference is that in health care services, turnover is high, material and premises are complex, and being operational is a matter of life and death in ED. (B) Implementation concept for an online employee onboarding tool: this 3D web app enables people to familiarize themselves with the premises and staff in a way that can bring properties sometimes even more effectively than in real life. For instance, to strengthen extraprofessional links, permanent staff allowed us to add some of their own personal anecdotes. We discovered that team members who had known each other for years deepened their bonds by discovering many personal aspects that they had never taken the time to share.

Staff turnover is a critical issue for most health departments [1,3,4], especially in EDs. These services operate 24/7, requiring personnel to be fully operational shortly after arrival. This constant demand increases pressure on onboarding processes and planning. High turnover in EDs stems from several factors, for example, intense workload, emotional fatigue, irregular hours, and the transitory nature of certain positions, which are linked to training periods or natural career progression. In addition, the ED at Lausanne University Hospital (CHUV) plays a leading role in clinical and postgraduate training for students, residents, and newly hired professionals. Many of these individuals are only present for a few months, for example, during postgraduate medical training. These short rotations contribute significantly to the department’s high turnover and the need for frequent onboarding and adaptation—a context and issue illustrated in Figure 1.

Unfortunately, in terms of education and training, the ED overcrowding does not always allow for suitable integration of these staff when they arrive in the department. The “permanent care” mission also complicates traditional face-to-face training, as some of the ED teams are on duty, whereas others are on recovery or night shifts. It is therefore important to find innovative solutions to improve team information, if possible, remotely and unplanned. The expectations of the new generation in terms of modern training also require new training modalities. Recent literature suggests that immersive and gamified environments may improve engagement, memory retention, and emotional well-being [5-7], yet few implementations offer both technical scalability and pedagogical adaptability.

The level of knowledge that must be quickly reached is important, justifying the necessity of finding solutions to improve training. One of the difficulties for trainees and new employees is to quickly understand the ED architecture, functional organization, and the principles of patient prioritization. The organization of an ED plays a key role in its operation, and a deep understanding of it is essential for teamwork and efficiency. The architectural structure of the service, functional organization, patient triage, and flow management is highly codified in the ED and needs to be learned quickly. The department’s missions involve knowing not only the different care areas, resuscitation rooms, and patient flow management from the waiting room but also the locations of resuscitation or medical equipment or even emergency exits and alarm systems (eg, fire and assault).

### Problem Statement and Work Objective

Despite advances in health education technologies, there remains a significant gap in the availability of autonomous, editable, and web-accessible onboarding tools that can address the operational demands of high-turnover hospital units. Most existing solutions require costly technical maintenance, lack content flexibility, or fail to offer meaningful interactivity for new hires. This article explores a solution to simplify the issue of frequent integration of new staff in the ED context using an online application that prospective employees can practice before arriving on site (Figure 1B). This work presents the design, development, and formative evaluation of a dual-component solution: (1) a web-based 3D gamified simulation of the ED aimed at improving staff onboarding; and (2) a companion quest management application enabling permanent staff to autonomously update and adapt onboarding content without IT intervention.

### Research Questions and Hypotheses

We posed the following research questions (RQs) to better understand the relevance and usability of this approach and derived 2 main hypotheses:

- RQ1: Can a browser-based gamified simulation improve perceived onboarding efficiency and engagement among ED staff?
- RQ2: Can permanent staff members, with minimal training, autonomously maintain and update onboarding content using a dedicated quest management tool?
- Hypothesis 1: The gamified simulation will be perceived as useful, engaging, and appropriate for onboarding by most health care professionals, as measured by usability and acceptability metrics.
- Hypothesis 2: The companion quest management tool will be usable and effective for nontechnical ED staff, enabling sustainable onboarding content maintenance.

### Gamified Simulations

Simulations in health care are essential to improve the skills of health care professionals [8]. They allow the replication of varied and complex clinical situations, fostering practical and secure learning. Gamifying these simulations makes the training more engaging and motivating [9]. By incorporating game elements such as points, levels, and rewards, participant involvement is increased, which enhances knowledge retention and skill mastery. Thus, the purpose of gamified simulations is to optimize training by making it more interactive and stimulating. In the field of health care, recent research includes a large set of aspects, such as simulation, 3D environments, gamification, serious gaming, patient sorting, urban reconstruction, and scripting [10].

In the context of urban interior and 3D visualization, Joy and Christhu [11] proposed a digital 3D modeling system for preconstruction. This study is important because it establishes a framework that explores the complexities of 3D architectural visualization in a real-time engine, which we have achieved. The concept was to enable a visual experience with a higher level of user interaction and then deploy it through virtual reality (VR). In our case, we did not choose to deploy the simulation in VR, as we wanted any future employee to be able to use it with a simple web browser. In the same domain, Chen and Feng [12] provided an original procedure to autogenerate buildings using cellular automata. Focusing more on the hospital, De Oliveira [13] proposed a platform composed of a set of tools that provides a patient-oriented modeling process, enabling the running of a simulation experiment showing possible outcomes in a 3D virtual scenario in a hospital environment, which is close to the problem raised in our proposal. In 2000, the same authors also presented a 3D visual simulation of hospital admissions [14]. Staying within the hospital field, 15 years later, de Lim et al [15] described a virtual environment for the learning process of simulated clinical cases for students and professors.

Concerning simulation, emergency, and gamification-related approaches, Gobron et al [16] proposed in 2019 a picture-based serious game to train nonmedical people in emergency situations. This simulation also included training to answer emergency calls. Yang et al [17] also provided an interesting review of the literature relative to crowd evacuation, which is a critical issue when simulating a public environment. A little further away, Shapiro et al [18] determined how simulation-based clinical team training improves performance when added to a didactic program, demonstrating that a gamified simulation can be efficient. A study by Zielke et al [19] explored the potential of VR and augmented reality for the development of a virtual patient in a communication practice—ie, managing patient emotions.

Recent (2021∼) works demonstrate the actual benefits of simulation in medical education: Krishnamurthy et al [5] provided a systematic review of what is meant by gamification in the health field; Gue et al [20] surveyed residents to investigate the impact of gamification in emergency medicine residency training, and Van Gaalen et al [21] proposed a review focusing on empirical evidence for the effectiveness of gamification approaches and the theoretical justification for the application of the chosen game attributes. In the field of gamified applications and employee training, Larson [22] proposed a literature review on serious games and gamification in the corporate training environment. The latest noteworthy publication is probably the work of Stoicu-Tivadar et al [23] presenting a virtual hospital architecture called eduCRATE.

Recent narrative reviews have highlighted the increasing use of gamification across various branches of medical education, including dermatology, where gamified tools have been used in both student training and patient engagement [24]. This broader trend reinforces the legitimacy and adaptability of gamification mechanisms in health care and supports their relevance as a pedagogical and communication strategy in clinical contexts.

### Onboarding Strategies

Several institutions have implemented structured onboarding initiatives to mitigate the impact of staff turnover, particularly among newly hired health care professionals. For instance, a 187-bed community hospital in Washington, DC, deployed a 10-element onboarding improvement program based on staffing statistics, exit surveys, and best practices identified in the literature. The intervention notably included reinforced managerial interactions and peer mentoring. The results were significant: new-hire turnover decreased from 39.1% to 18.4%, and overall hospital turnover dropped from 18.2% to 11.9% (Z=−2.06; *P*=.04) [4]. These findings underscore the value of standardized communication-focused onboarding processes supported by cross-departmental collaboration and executive commitment.

Health care professionals face constant emotional strain and demanding work conditions, generating a growing need for integrated emotional relief mechanisms within their professional routines. Recent studies have highlighted the dual function of gamification—not only as an engagement tool but also as an emotional outlet. Johnson et al [6] demonstrated that game-based elements can enhance motivation while positively influencing well-being. More recently, in 2024, Tu and Lee [7] reported a significant reduction in stress levels among shift-work nurses using a gamified intervention platform. The same year, Lehtoranta et al [25] similarly emphasized the positive impact of gamification on employee satisfaction and mental health. These findings suggest that, in high-pressure clinical environments, the inclusion of a simulation that blends serious objectives with playful dynamics can support both training effectiveness and psychological resilience. As such, gamified systems offer a promising response to the emotional and cognitive demands experienced by health care staff in daily practice.

In terms of 3D simulations applied to health, Rash et al [26] proposed in 2024 a prototype of a 3D virtual simulation emergency room environment for interprofessional team training. Unfortunately, this prototype does not include any quests or motivating interaction for field teams, nor does it allow staff to modify the scenarios. Moreover, only oral feedback from a dozen participants was presented. By far, the closest publication to our approach is that of Shi et al [27], proposing the simulation of part of a hospital department. The similarities with the current proposal include a 3D simulation with quests. What we found especially interesting and positive in this approach were several features: nonplayer characters (NPCs; also called agents) can move; however, it is not specified how; player inventory system is a strong plus; fallback toward a local database if online connection with the cloud is off; and their minimap has the possibility to add markers, which can be useful when discovering a new place. Still, unfortunately, the fundamental weaknesses (that our proposal solves) are as follows: Their app runs locally on PC; it cannot have the flexibility of a web-based app, and current graphics texture is heavy (texture), which strongly restricts the portability of online deployment; user to agent/NPC interactions only consist of dialog (eg, no question/answer); no comparative visualization with the real views of the hospital; no login user/password access, which implies security weaknesses; lack of data validation; new quests only are added using a Microsoft Excel local file; and adding assets (eg, new permanent staff members) is not possible.

## Methods

### Overview

This section presents the objectives, application deployment, key aspects of system design, and software architecture. While inherently technical, these details are crucial for ensuring effective collaboration between medical and IT teams, a challenge that has led to the failure of many similar projects. By outlining the underlying architecture, development choices, and integration mechanisms, we aim to provide both hospital medical staff and IT departments with the necessary insights to deploy, maintain, and adapt similar solutions to evolving needs.

### Objectives and Design Process

A hospital and engineering consortium was set up to take up the challenge of developing an application that would solve—at least in part—the problem of rapid integration in an ED context. In collaboration with the CHUV ED and the University of Lausanne, a digital innovative solution was developed to ease the integration of new employees. The design and development of the simulation platform took place over a period of 7 years, with a notable interruption due to the COVID-19 pandemic. Throughout this process, we adopted an iterative and collaborative design framework, working closely (every month) with the ED’s medical staff. This continuous involvement ensured that the evolving needs, constraints, and pedagogical expectations of the department were consistently addressed. Owing to the inherent nature of the project, the actual target group—that is, newly arriving staff—was, by definition, not accessible for testing during development. However, the permanent team provided valuable feedback from the perspective of onboarding processes and acted as proxy users, offering realistic input on usability, content relevance, and scenario design. This sustained collaboration between technical developers and medical professionals laid the foundation for a solution tailored to the real operational dynamics of the ED, thus supporting its long-term sustainability and practical adoption.

**Figure 2.**
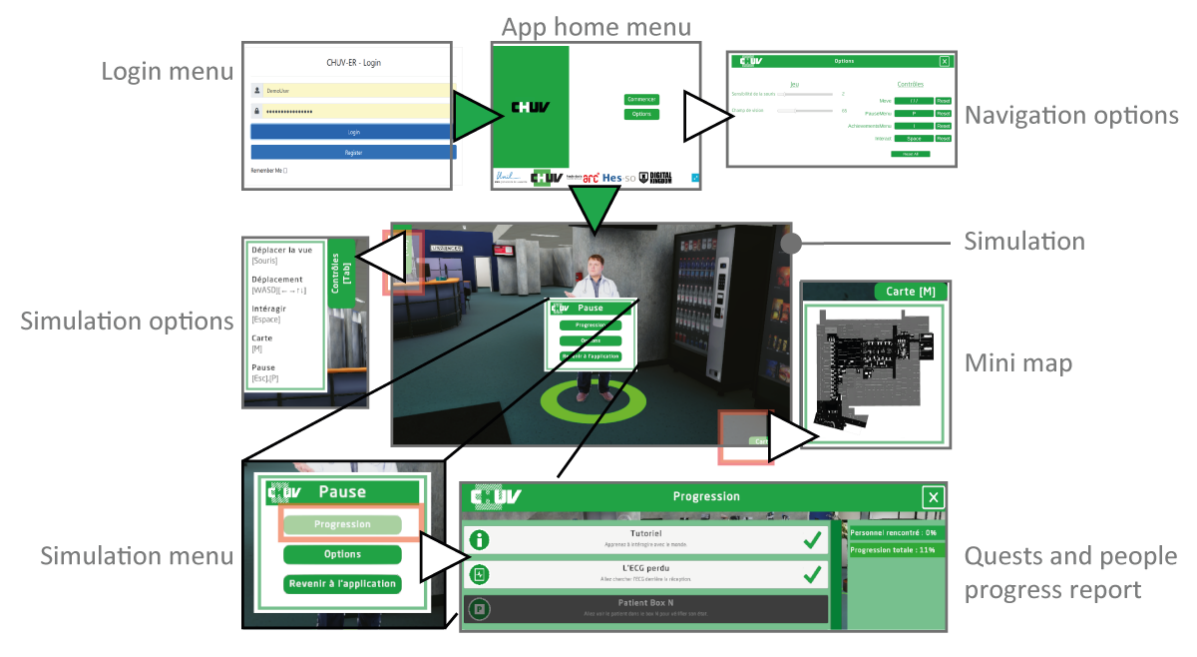
Illustration of gamified simulation windows. To reach the 3D simulation, follow the green arrow steps; 5 other windows are for the user management of the navigation and the quests’ follow-up (white arrows highlight these options).

The digital solution was based on an interactive 3D application (illustrated in Figure 2), which provides future staff members with virtual immersion in the hospital premises, as well as interaction with simulated staff and patients. To achieve the solution proposed in this paper, we had certain basic criteria and others that were discovered as we went along:

1. Challenges: to ensure that the locations are realistic (geometrically, spatially, realistic camera movements and angle of view, colors and textures of floors, ceilings, walls, furniture, posters and main instructions, and medical equipment); to provide a solution that is easy to use in terms of the interface and adapted to the user’s profile; and to supply serious, rewarding, and fun encounters with the NPCs.
2. Constraints: to develop a lightweight 3D application for a convenient online access, to ensure security with user login and password, and to enable easy-to-edit quests without the need for IT or 3D specialists.

**Figure 3.**
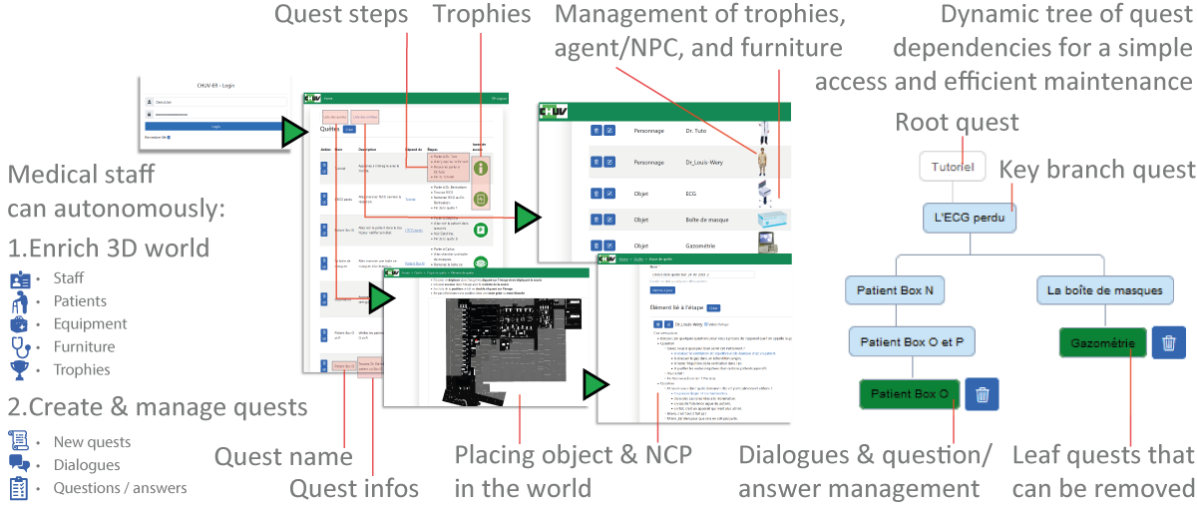
Use of the application to manage quests. Medical materials or any object as well as new characters can be added via images as “billboards” (ie, PNG images with transparency). The scale of these images is managed by the following ratio: 500 pixels for 1 m. Once these assets are uploaded, the user can define a new quest assigning various objects and virtual agents. An optional personified trophy can also be associated for each quest—if not, a default picture will be assigned. Each quest includes dialogs, questions, places to go, and objects to find, which implies the use of map coordinates as shown in the figure. NCP: nonplayer character.

The key element of this application lies in its gamified approach, which transforms integration into a fun and engaging experience. Through a variety of quests, such as discovering locations, meeting virtual colleagues, or solving specific tasks, new employees are guided in their learning while being encouraged to actively explore their environment. To ensure the longevity and flexibility of this solution, a second application was developed for permanent staff (see properties in Figure 3). This application enables in-house teams to modify and add quests, rewards, and other simulation elements without requiring the constant intervention of the technical team. This approach ensures a rapid adaptation to the frequent changes observed in hospital services while preserving the quality and consistency of the induction experience for new employees.

Two applications were developed: one for new employees and one for the permanent staff to manage the quest system (Figure 3), without having to go through the IT department or a costly external service provider. The current application provides an unlimited number of quests, where each of them can also have implicit dependencies and subquests. Within each quest, five types of tasks are defined: (1) go to an area—mainly to discover the relatively complex topology of the ED; (2) dialogs from basic virtual agents—mainly associated with personal anecdotes; (3) basic conversation with virtual agents—usually part of a quest; (4) search for an object or a person, for example, to understand professional functions and/or duties; and (5) getting a multiple-choice question (2-4 options) and answering it, for example, for patient sorting.

### 3D Application for New Employees

The end user application is a 3D interactive simulation that models the ED. This application was designed using Unity Engine version 2021.3 and is based on the Universal Render Pipeline. Thanks to this engine, a multiplatform deployment was possible in a web environment. Deploying a real 3D application as a web page (ie, using WebGL) offers unparalleled accessibility and scalability. Unlike traditional software that requires installation, a WebGL-based solution runs directly in the browser, making it independent of both hardware and operating system. This ensures seamless deployment on PCs, tablets, and even smartphones, without compatibility concerns. In addition, updates are centralized, eliminating the need for manual installations while providing real-time access to the latest version, a crucial feature for dynamic hospital environments. This makes the application more accessible, easy to maintain, and avoids time-consuming local installations that could lead to security breaches. Once users successfully authenticate themselves on the login page, the application conveniently loads in their web browser. Therefore, without any constraints, an immersive gamified simulation in first-person view can be used at any time and from any location with a common use network.

One of the main challenges was to produce a realistic 3D simulation of such a complex building, which could be rendered on a simple web browser in real time. For this kind of online use and considering the topology (Figure 3), in addition to improving the frame rate (frame per second [FPS]) and the visual quality, we had to reduce the overall application size for a faster download and ensure that the application data were not evicted from the browser cache mechanism. To achieve these optimization goals, we applied the following procedure:

- We simplified heavy graphical meshes (ie, geometric modeling) from the hospital scene and removed pieces that were never visible from the player’s point of view (eg, elements under beds or within inaccessible areas).
- We reduced the size of all environment textures to their minimal acceptable size (64, 256, 512, etc) and enabled texture crunch compression option when available.
- As most objects are static in the scene, we used the static batching feature of Unity to improve the FPS as well as the light so-called *baking* feature to improve the visual quality with a lower impact on FPS.
- As our tests on occlusion were not demonstrating the expected improvement, we did not use this optimization feature even if it theoretically seemed adapted.
- Finally, to embed the application in our web page, we simply used the Unity WebGL exporter and made sure to deactivate the exception support as advised by Unity to gain a few additional FPS.

These optimizations allowed us to achieve an acceptable result in terms of size, speed, and visual quality (ie, 89 MB load and 25-35 FPS) for our medium tier computer: CPU Intel(R) Core (TM) i7-7660U CPU @ 2.50GHz; GPU Intel(R) Iris(R) Plus Graphics 640; 8 Go of RAM. Within the study, we believe that an interesting track would be using an incremental download mechanism using asset bundles to have a better “Start Render Time” for the application.

### Quest Management Application

**Figure 4.**
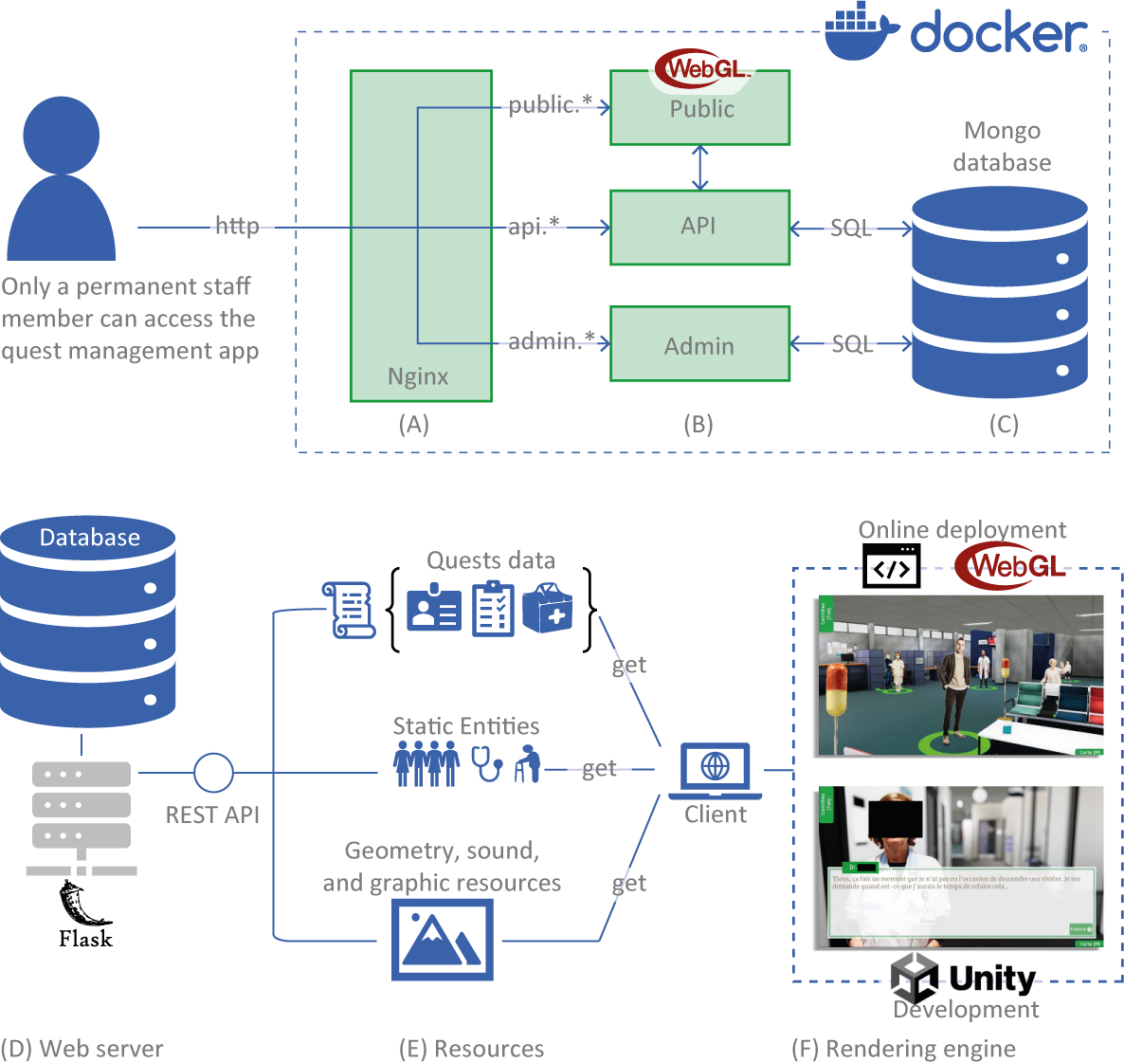
Technical illustration of the quest manager application. Every service runs inside a Docker-composed environment. A virtual network is created to ensure seamless communication between the 3 main components: (A) Nginx (an open-source software for web serving) orchestrates the access to the 3 microservices using a reverse proxy mechanism; (B) 3 microservices are deployed as Docker containers and the web framework used is Flask; (C) the database is deployed along the different services allowing data storage and we used *MongoDB* as database system. Data stored and managed in the quest management app: from (D) database and web server, through (E) types of resources, and up to the Unity-based gamified simulation (F). API: application programming interface; SQL: structured query language.

For security reasons, the quest management app illustrated in Figure 3 can only be used by permanent staff members. Its implementation uses a microservice-oriented architecture with Docker containers (Figure 4). It consists of three main microservices: (1) the public microservice, which serves as the frontend for end users and provides access to the Unity application with WebGL deployment; (2) the application programming interface (API) microservice, which furnishes a RESTful API (ie, API that conforms to the constraints of REST architecture) for the WebGL application to retrieve data from the database; and (3) the Admin microservice, which serves as the core infrastructure component, helping staff members in easily managing quest command lines for the game, including creation, reading, and editing functionalities.

The access to the different microservices is orchestrated via Nginx. A reverse proxy mechanism is deployed to simplify access to diverse services by using a standardized nomenclature.

### Link Between Both Apps

The application communicates with the server database via a REST API, retrieving information in JSON format (see Figure 4). This information includes graphic resources, character and interactive object placements, dialogs, and quest details, such as task sequences and success conditions. When the 3D simulation launches in the web browser, this information is transmitted, and the entities and quests are dynamically instantiated within the simulation, enabling dynamic creation of quest elements or entities.

### User Tests

Before any deployment, we needed to test with people who did not know the project, but who were familiar with the site: that is, current permanent staff members. With this in mind, we took the initiative to carry out 2 preliminary tests. Data were collected using anonymous Google forms: (1) administrative and explanatory information before testing the apps and (2) sets of questions relative to the appreciation of different aspects of the apps with optional suggestions for improvements. The questionnaires were adapted from validated usability and acceptability instruments, and data were analyzed using standard descriptive statistics, ensuring methodological robustness despite the preliminary nature of this study.

The user tests were anonymous with a double-blind protocol to assess performance and user experience objectively. In this protocol, neither the participants nor the evaluators were informed about the test conditions to reduce potential biases. In addition, the evaluators collecting data and analyzing results remained unaware of the identity or the experimental experience of the users.

### Ethical Considerations

#### Ethics Review

This study assessed the feasibility of a digital simulation platform through anonymous surveys of adult health care professionals. No patients, medical records, or personal or health-related data were involved. According to the Swiss Human Research Act (*Loi relative à la recherche sur l’être humain, LRH, RS 810.30*) and the guidance of the *Commission cantonale d’éthique de la recherche sur l’être humain du Canton de Vaud (CER-VD*), such feasibility studies with anonymous professional staff do not fall within the scope of mandatory ethics committee review [28,29]. Consequently, no formal ethics board application was submitted.

#### Informed Consent

All participants were adult health care professionals who provided verbal informed consent before participation. They were informed of the voluntary nature of their participation, the absence of personal or health-related data collection, and the anonymity of their responses. Written or electronic consent was not required because the study posed no foreseeable risks and collected no identifiable or sensitive information.

#### Privacy and Confidentiality

All data were fully anonymous and deidentified at the point of collection. Surveys were conducted using Google Forms with IP tracking disabled. In compliance with institutional policies and the General Data Protection Regulation, no personal identifiers were stored, and only aggregated anonymized results were exported to secure institutional servers (HES-SO, UniL, and CHUV).

#### Compensation

No compensation, financial or otherwise, was offered or provided to participants for their involvement in the study.

#### Image and Participant Identification

All rendered illustrations included in the manuscript were designed to anonymize staff members. No recognizable individual is visible, and facial features, names, and personal identifiers have been either removed or replaced with nonidentifiable placeholders. Few pictures are not anonymized because they have been autogenerated and do not correspond to real, permanent people.

## Results

### 3D Environment and Staff Representation

**Figure 5.**
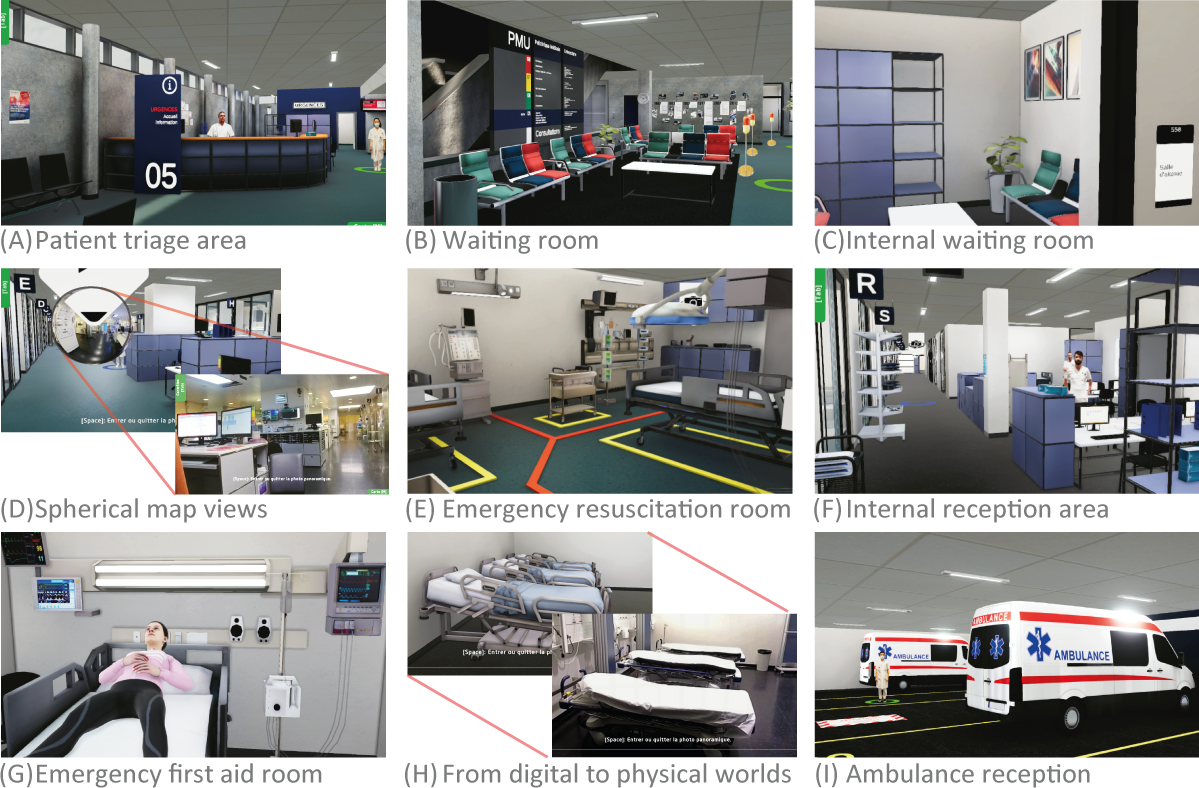
Area examples of gamified simulation: (A) main reception of the emergency department (ED); (B) main waiting room; (C) 1 of the 4 waiting rooms, even the table is visible in the physical world; (D) a strange sphere is visible at every intersection of the ED: it represents a real spherical photo of the premises to increase the connection between the physical and digital worlds; the user can enter it simply by approaching and looking at it, and leave it by clicking on the Esc key; (E) this vital ED room is equipped with numerous pieces of equipment and is located right next to the emergency entrance; (F) representation of a pivotal area for the emergency teams: the administrative area for personnel management; (G) one of the numerous first and second aid ED rooms; (H) comparing area with physical place; and (I) reception area for patients arriving by ambulance.

The results of the gamified simulation mainly consist of 2 categories: a 3D rendering environment where new employees can discover the working place in a 3D real-time rendering (Figure 5) and permanent staff members’ graphical and dialog representations.

**Figure 6.**
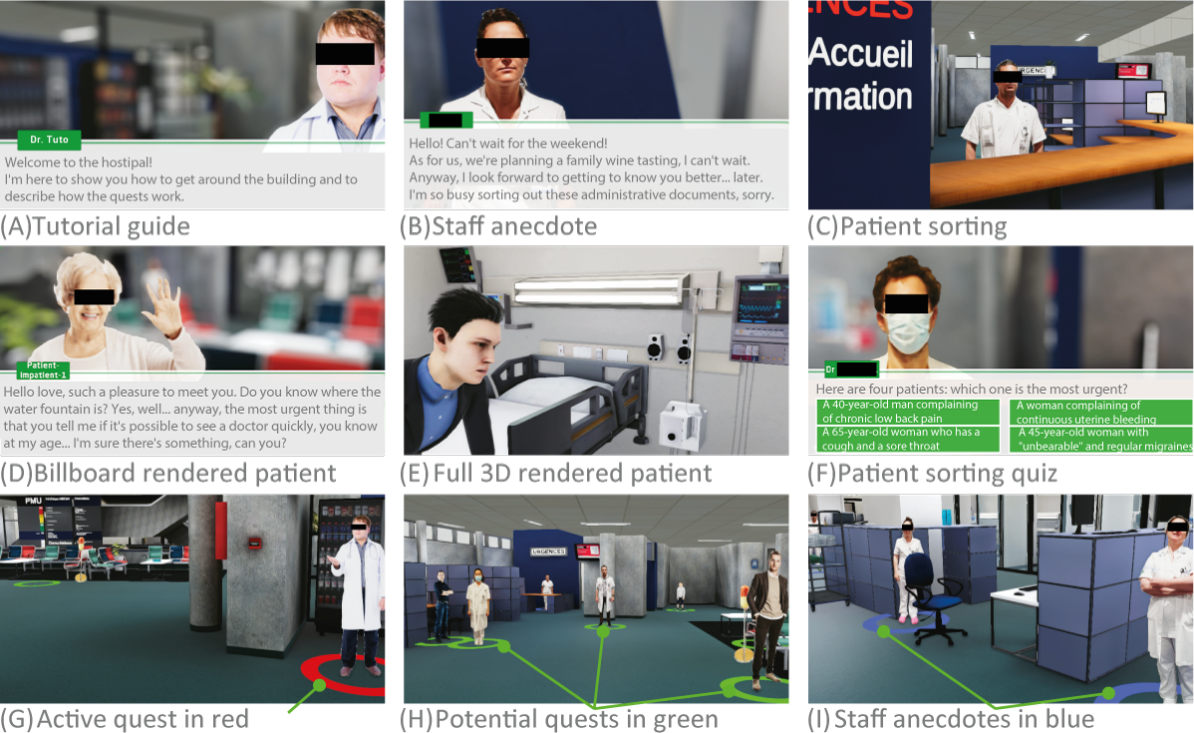
Examples of encounters: (A) the first interaction users will have a tutorial proposing first steps in the 3D environment; (B) an example of an anecdote; (C) the main welcome desk where you can train patient sorting; (D) interaction with a patient rendered with a billboard; (E) an example of a patient rendered in real 3D; (F) a quiz on patient sorting; (G) color codes indicating how to interact with agents: green, a quest can be activated; red, a quest has been activated; blue, an anecdote can be read; (H) a set of multiple interactions for quests; and (I) a set of multiple anecdotes that can be read.

**Figure 7.**
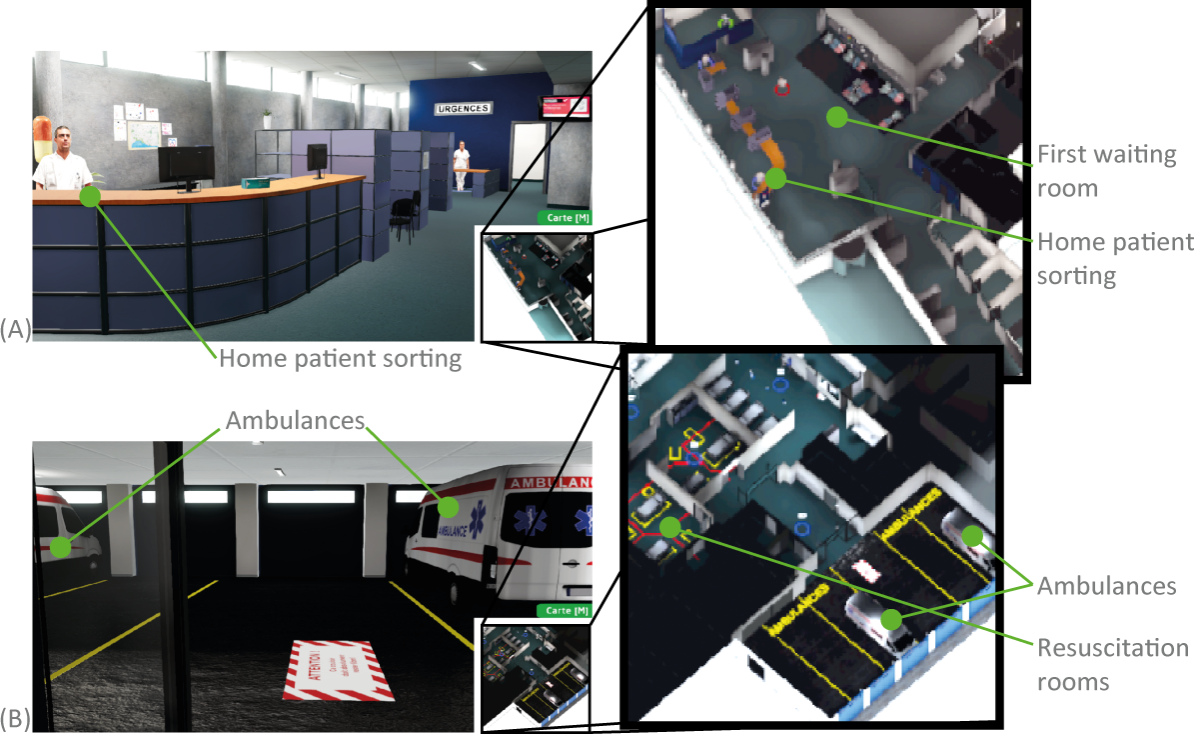
Illustrations of the minimap. Two locations (A and B) where we activated the ‘M’ key to bring up the minimap in the bottom right-hand corner of the screen; their respective enlarged views are shown on the right.

Figure 6 illustrates 9 possible staff interactions. Three types of characters are represented in this gamified simulation: permanent team members (as billboards only) and patients—as billboards or in full 3D. Billboard characters are those that can be changed into quests. This simple billboard representation presents 3 advantages: (1) it is a simple textured rectangle with transparency; therefore, it is impossible to make it any lighter for the online application; (2) anyone can take a photo and clip it with the automated clipping, which is a common feature on smartphones; (3) changing the scale (100 pixels for 1 m) and importing an image into the quest management application are within the reach of non-IT staff. Discovering such a large environment, with almost a hundred elements, can seem like a labyrinth. To help the user on the matter, we have added a minimap view that can be displayed by pressing the ‘M’ key, as shown in Figure 7. Interaction with agents (ie, digital characters) takes place through dialog with the possibility of associated questions and answers. Quizzes can be set up, and missions can be easily described.

### Results of the Preliminary User Tests

#### Overview

As there were 2 applications, we submitted 2 user tests (n=37 and n=9) and corresponding 2 questionnaires to the ED staff. We needed to know the opinion of the permanent staff as soon as possible for final parametrization of the prototypes (and therefore before conducting the large survey of naive users that we are preparing for Fall 2025). The profile of the respondents based on the information extracted from the survey and the plant’s stats are illustrated in MultimediaAppendices 1  2.

#### 3D Web App User Test

We conducted a survey regarding the 3D gamified simulation with 37 naive participants (all permanent staff members) and gathered results on 5 main criteria along with appreciation percentages. Providing personal anecdotes was considered motivating by 79% (29/37) of the respondents. To the question, *Does the app allow you to meet and get to know the permanent employees a little?*, 73% (27/37) of respondents believe it has potential; and to the question, *Does it help to get to know the equipment better?,* 82% (30/37) of the respondents agreed on it. The topology of the ED received 90% (33/37) positive answers with the lowest SD, indicating strong unanimous agreement. Finally, for the question *Do you believe this app is useful for new employees?*, 81% (30/37) of respondents found the app useful for new employees, suggesting more varied opinions among respondents—ie, the highest SD.

#### Maintenance Web App User Test

The online software used to create and manage quests was also evaluated with 9 participants (permanent staff members as well, three of them already aware of the 3D web app but not of the maintenance software), as shown in Multimedia Appendix 2.

The results are a little more varied but still positive: 91% (8/9) of respondents praised the modification possibility of the 3D simulation without the help of the technical staff. The construction of quests to discover the workplace and the people involved was also highly appreciated. The quests seemed to be relatively easy to build (77% approval), still with an elevated SD of 35%. Finally, on the question of whether scores and trophies were a source of motivation, responses were moderately positive, with 70% approval.

## Discussion

### Principal Findings

First of all, the large difference in participant numbers between 37 and 9 can be explained by the fact that the quest creation and management application can only be used by key people accredited by the ED. Given the long-term, interdisciplinary nature of our development effort, we believe that 5 aspects merit focused discussion. First, estimating the practical impact of our approach is essential to demonstrate its relevance in addressing real-world constraints, such as time, cost, and staff turnover. Second, the real-time 3D simulation and its gamified design form the technological and pedagogical core of our solution, directly shaping user engagement and memory retention. Third, the quest management application represents a crucial operational tool that empowers nontechnical medical staff to adapt and extend the simulation autonomously, ensuring sustainability. Fourth, sharing the challenges and insights from the multiyear deployment process provides valuable guidance for other teams pursuing similar innovations in hospital environments. Finally, exploring the generalizability of our platform to other departments helps establish its broader applicability, reinforcing the value of the framework beyond the ED context.

Implementing a gamified simulation similar to the one presented in this project has significant practical implications for a medical department, in terms of both costs and benefits. In financial terms, the development of the project required around 3000 engineer hours spread over 8 years. This relatively long period can be explained by starting from scratch, coordination between interinstitutional partners, and the prolonged interruption due to the COVID-19 pandemic. However, this initial investment can be justified by the potential gains offered by the solution once deployed.

In terms of benefits, the impact on team time and workload could be major. With a 100-person department with an annual turnover rate of 22%, which implies the integration of approximately 22 new employees each year, the estimated 30% to 40% reduction in integration time would save theoretically 15,400 hours per year. These time savings can be reallocated to activities directly linked to patient care. Similar results have been observed in previous studies [4,30-32], such as the implementation of a 10-element onboarding program in a community hospital, which reduced overall annual turnover from 18.2% to 11.9%, and new-hire turnover from 39.1% to 18.4%. Additionally, structured onboarding processes in EDs have been associated with improved operational efficiency, as evidenced by a significant decrease in patient door-to-discharge times.

Another significant benefit is improved productivity and quality of care. By enabling new employees to quickly familiarize themselves with the premises, equipment, and colleagues, the simulation reduces the stress associated with taking up a new post and accelerates their skills development. This promotes better coordination between teams and reduces the risk of errors due to lack of preparation or familiarity. Ultimately, the solution not only helps streamline onboarding processes but also improves operational efficiency and improves the patient’s experience, even in an environment as demanding as the ED. Finally, the political dimension, the motivational aspect, highlights the department’s ability to lead innovative and modern projects, enhancing its appeal and attractiveness.

### 3D Gamified Simulation

Overall, it seems that the simulation was positively received, especially in terms of helping users get acquainted with the equipment and the actual working environment. However, there may be some variability in opinions about its usefulness for new employees. Claims about potential time savings, efficiency gains, and improved team integration should therefore be interpreted as plausible expectations supported by prior literature. Still, they remain hypothetical until validated through longitudinal or performance-based evaluations with actual newcomers. This feedback can be valuable for further refinement and improvement of the simulation. As for the deployment, we chose to deploy the apps on a gamified browser-based, real-time 3D simulation to increase immersion and realism with a relatively realistic environment (texture, lighting, topology) so that the gamified aspect potentially allows greater engagement of new employees and links long-term memory with a certain degree of emotion and web deployment means that the application can be accessed from anywhere, PCs, tablets, or even smartphones.

However, each of these properties can be improved. First, health departments often evolve; for example, rooms are reorganized and walls may be removed or built. These aspects are not currently supported by the quest maintenance software, implying that annual 3D updates should be required. Second, even if we had positive feedback from the first trials, professional-based quests should be tested; furthermore, current testers were permanent staff who know well the ER, so a large-scale user test with adequate target profiles must be set up and analyzed. Third, we did not test the app on low hardware configurations; it is necessary to study the limits in terms of hardware setup and graphics support (eg, resolution).

### Quest App and Corresponding User Test

As presented in the previous section, the quest management application is functional, and the staff who tested it are enthusiastic. Note that these are the same employees who will be using it—not the new employees who will be using the online 3D simulation. The current ergonomics, due to a lack of means, could only be modest, even involving the (admittedly simple and automated) writing of XML code to implement the questions. Major efforts are required to make this application more user-friendly and ergonomic.

### Two Larger-Scale User Tests

The abovementioned user tests were needed in the development process (see section Reaching Actual Deployment) but are not sufficient for the final deployment because we do not know the actual impact of this gamified simulation on actual end users. To address this issue, we are now scheduling 2 large-scale user tests: one with first- or second-year students (n>100), and 6 months later, one with the ones visiting the actual ED.

### Reaching Actual Deployment

**Figure 8.**
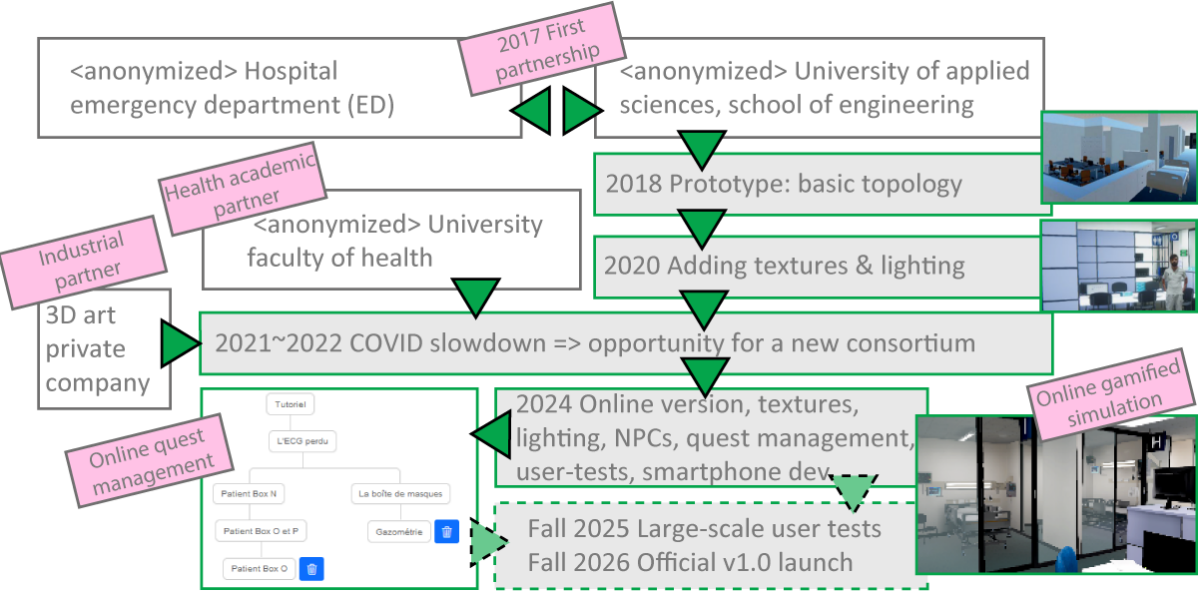
Design process of the project. This figure illustrates the 8-year design process of this project, including the consortium of the project and versioning progress during the last 8 years, with the first prototype being presented in 2018: with 7 types of partners—engineers, researchers, technicians, managers, doctors, specialists, and nurses—from quite distant fields. The financing and organization of such a project requires careful planning and strong communication.

The context for deploying this kind of application—involving 4 institutes—in a hospital is atypical, which is why we believe it is important to present the steps we took to get there. As shown in Figure 8**,** we have been building this gamified simulation for about 8 years now and are in the fourth version of the project, started in 2017 [33]. Setting up an innovative project with well-defined needs, with a skilled and motivated team, with sufficient funding, is unfortunately not enough for a health care project to be implemented and really used: upstream, the institution’s policies at both management and technical (IT department) levels must be considered. Indeed, IT and health imply important security issues, and when patient information is stored in the same building, an outcry can easily arise. Security on hospital servers is of paramount importance. To solve this problem, we have dedicated an external server with no connection (software or hardware) to internal IT services.

### Generalizability of the Tool Beyond ED

Although this simulation was specifically designed for the onboarding of ED staff, the architecture of the system was deliberately built with adaptability in mind. The core components of the platform—modular 3D mapping, customizable quest creation, and a web-based interface—enable the deployment of similar simulations in other hospital departments, even those with markedly different layouts or protocols. For example, departments such as radiology, intensive care, or surgery, each with their own spatial organization and workflows, could benefit from tailored versions of this system. The quest management application is designed to be fully editable by internal medical staff, without technical expertise, allowing adaptation to diverse professional roles, equipment, and team structures. Moreover, the interaction mechanisms (eg, dialogs, location-based tasks, quizzes) are generic and can be aligned with various onboarding objectives. Future developments will include tools for modular editing of building geometry (ie, nontrivial development where artificial intelligence will play an invaluable role), enabling even greater adaptability to departments undergoing spatial reconfiguration. While initial testing has focused on ED-specific challenges, the fundamental design supports scalability and generalization. A pilot adaptation for another department is currently under discussion to validate this potential. Ultimately, the platform aims to provide a reusable framework for immersive onboarding across the broader hospital ecosystem. This scalability is not only theoretical but also structurally embedded in the system’s design: the geometry of any department can be replicated, whereas local staff are empowered to create department-specific scenarios, personnel representations, and equipment, all without IT support.

### Tracks for Further Research and Perspectives

#### Overview

As introduced in the Discussion section, many aspects can be improved to make the 3D simulation even more realistic, especially the textures and lighting (eg, with real-time ray-casting). Still, we do not think the priority should be on graphics, but rather on content (eg, quests). In addition, before any actual deployment, a study of the real impact/benefit needs to be carried out, not with current employees, but with so-called naive people (eg, medical or nursing students). We propose the following short- and long-term perspectives.

#### Short-Term Perspective

The first task to be improved is relative to the quest management app. Indeed, its ergonomics need to be adapted because end users will be medical staff members; for instance, the current ways to design questions are based on XML coding: this should be replaced with point-and-click drop boxes. Furthermore, in terms of hardware, we are currently looking into the design of a dedicated server that would have no connection with the hospital’s servers and would allow total security of patient data.

#### Long-Term Perspective

The primary goal is actual deployment. To achieve this, we will have to use feedback on the above-described large-scale user test and develop the corresponding improvements for the official deployment. One of the aspects we need to study is the impact and effectiveness in terms of time savings not only for health departments but also for the human characteristics and satisfaction of new employees. At an even longer term, we would like to set up a system that allows permanent medical staff to easily modify furniture and equipment in wards. The idea is to give a solution that simulates the often-necessary rearrangements in hospital institutes. In this context, as suggested in 2025 by Lindner and Leutritz [34], we would also like to provide VR deployment for a better understanding of real-life working situations (eg, reorganize a medical room, check that roll-away beds can be moved around the building properly). In addition, it would be interesting to integrate a mechanism for an easy updating of the building geometry; for instance, splitting in parts or the possibility of adding a semiautomatic 3D scan for new walls or openings. This last point is particularly important because it would enable similar gamified simulations to be deployed with much less effort for different health care services. It would also open a much broader vision, for example, on a hospital-wide scale.

Beyond the scope of this study, further research is critically needed to assess the long-term effectiveness of gamified onboarding solutions in health care. This includes evaluating their impact on staff retention, clinical performance, and team cohesion across various medical settings. Comparative studies across institutions with different cultural and organizational structures would help determine the generalizability of such tools. Furthermore, economic evaluations are necessary to analyze the balance between initial development costs and savings in recruitment and training. Finally, a better understanding of how specific game elements affect user motivation and engagement could help optimize simulation design based on department profiles and learner characteristics.

The following Table 1 summarizes the aspects we wish to implement to improve the perception of real adoptability.

**Table 1.** Improving the perception of real adoptability.

Dimension	Current state	Improvement path
Usability	Web-based app tested on medium hardware	Evaluate on low-end devices/older browsers
Content flexibility	Quests editable via XML	Transition to point-and-click editor (planned)
Engagement	Positive feedback from permanent staff	Test motivation and engagement in naive users
Security	Hosted on external server	Consider audit by hospital IT/independent review
Deployment readiness	CHUV^a^ pilot completed	Prepare documentation for interhospital adaptation

a CHUV: Lausanne University Hospital.

### Conclusions

This paper is fundamentally procedural, focusing on the why and how behind the development of a gamified simulation for hospital onboarding, rather than an analytical assessment of its long-term impact. While the necessity for improved onboarding solutions in high-turnover health care environments is widely acknowledged, many similar initiatives struggle to succeed due to the gap between IT specialists and medical staff.

Our experience suggests that the failure of such projects is often due to a lack of structured collaboration and mutual understanding between these 2 domains. To bridge this gap, we have detailed not only the rationale but also the entire development process, ensuring that both medical and IT professionals can grasp the conceptual, technical, and operational aspects required for successful implementations. The technical sections of this paper are thus deliberately aimed at hospital IT departments, which play a key role in ensuring the feasibility and sustainability.

### RQs and Hypotheses

In parallel with this implementation perspective, the study also addressed 2 guiding research questions concerning (RQ1) the onboarding effectiveness of the gamified simulation and (RQ2) the operational autonomy enabled by the quest management tool. The preliminary findings provided encouraging support for both associated hypotheses: (H1) that the simulation would be perceived as useful and motivating and (H2) that permanent staff could manage onboarding content without IT support. While not yet a full-scale impact assessment, these results help establish a foundation for further empirical evaluation.

The close collaboration between the different partners has yielded an innovative dual app solution aimed at swiftly integrating new personnel. Through an interactive 3D application, new staff members can immerse themselves in virtual hospital environments, engaging with simulated colleagues and patients. The gamified approach of the application guides new employees through various quests, easing active exploration and learning. The development of a companion application for ED permanent staff ensures the solution adaptability to the dynamic nature of hospital departments while maintaining quality and consistency in the integration process. This solution not only streamlines the onboarding process but also enhances overall efficiency and effectiveness in the ED’s workforce integration efforts.

Moving forward, future work will focus on large-scale deployments and measurable outcomes, including onboarding time reduction, staff satisfaction, and long-term retention metrics. The hypotheses introduced here provide a structured baseline for those efforts, and their confirmation or refinement will be crucial in validating the generalizability and clinical value of gamified onboarding platforms in health care.

## Supplementary material

10.2196/72202 Multimedia Appendix 1 Results of the first user test. These charts present the resulting statistics of the first user test with n=37 using a 6-point Likert scale: (A) profiles of survey people: age, gender, and role; (B) the use of the main gamified simulation.

10.2196/72202 Multimedia Appendix 2 Results of the second user test. These charts present the resulting statistics of the second user test with n=9 (i.e. supervisors only) using a 6-point Likert scale: (A) profiles of survey people: age, gender, and role; (B) the use of the quest management: tool made only by key people accredited by the emergency department (ie, permanent and managing the department).

## Abbreviations

API: application programming interface
ED: emergency department
FPS: frame per second
RQ: research question
VR: virtual reality

## References

[1] Castle NG (2006). Measuring staff turnover in nursing homes. Gerontologist.

[2] Merçay C, Grünig A, Dolder P (2023). Personnel de santé en suisse – rapport national 2021: effectifs, besoins, offre et mesures pour assurer la relève. https://www.odasante.ch/fileadmin/odasante.ch/docs/Bildungspolitik/Versorgungsbericht2021/Obsan_CDS_OdASante_03_Rapport_national_2021.pdf.

[3] Dwesini NF (2019). Causes and prevention of high employee turnover within the hospitality industry: a literature review. Afr J Hosp Tour Leis.

[4] Kurnat-Thoma E, Ganger M, Peterson K, Channell L (2017). Reducing annual hospital and registered nurse staff turnover—a 10-element onboarding program intervention. SAGE Open Nurs.

[5] Krishnamurthy K, Selvaraj N, Gupta P (2022). Benefits of gamification in medical education. Clin Anat.

[6] Johnson D, Deterding S, Kuhn KA, Staneva A, Stoyanov S, Hides L (2016). Gamification for health and wellbeing: a systematic review of the literature. Internet Interv.

[7] Tu MH, Lee SY (2024). Effectiveness of gamification intervention platform on the work stress of shift-work nurses in the post-pandemic era. Stud Health Technol Inform.

[8] Abildgren L, Lebahn-Hadidi M, Mogensen CB (2022). The effectiveness of improving healthcare teams’ human factor skills using simulation-based training: a systematic review. Adv Simul (Lond).

[9] Dahalan F, Alias N, Shaharom MSN (2024). Gamification and game based learning for vocational education and training: a systematic literature review. Educ Inf Technol.

[10] Haubruck P, Nickel F, Ober J (2018). Evaluation of app-based serious gaming as a training method in teaching chest tube insertion to medical students: randomized controlled trial. J Med Internet Res.

[11] Joy E, R. A, Raja C (2024). Digital 3D modeling for preconstruction real-time visualization of home interior design through virtual reality. Constr Innov.

[12] Chen Y, Feng M (2022). Urban form simulation in 3D based on cellular automata and building objects generation. Build Environ.

[13] De Oliveira MJF 3D visual simulation platform for the project of a new hospital facility.

[14] De Oliveira MJF (2000). Planning for the Future: Health Service Quality and Emergency Accessibility.

[15] de Lima RM, de Medeiros Santos A, Mendes Neto FM A 3D serious game for medical students training in clinical cases.

[16] Gobron S, Chatelain S, Bolinhas C, de Oliveira DC A picture-based serious game to train non-medical people for emergency situations.

[17] Yang S, Li T, Gong X, Peng B, Hu J (2020). A review on crowd simulation and modeling. Graph Models.

[18] Shapiro MJ, Morey JC, Small SD (2004). Simulation based teamwork training for emergency department staff: does it improve clinical team performance when added to an existing didactic teamwork curriculum?. Qual Saf Health Care.

[19] Zielke MA, Zakhidov D, Hardee G Developing virtual patients with VR/AR for a natural user interface in medical teaching.

[20] Gue S, Ray J, Ganti L (2022). Gamification of graduate medical education in an emergency medicine residency program. Int J Emerg Med.

[21] van Gaalen AEJ, Brouwer J, Schönrock-Adema J, Bouwkamp-Timmer T, Jaarsma ADC, Georgiadis JR (2021). Gamification of health professions education: a systematic review. Adv Health Sci Educ Theory Pract.

[22] Larson K (2020). Serious games and gamification in the corporate training environment: a literature review. TechTrends.

[23] Stoicu-Tivadar L, Stoicu-Tivadar V, Berian D, Drăgan S, Serban A, Serban C (2014). eduCRATE--a virtual hospital architecture. Stud Health Technol Inform.

[24] Szeto MD, Strock D, Anderson J (2021). Gamification and game-based strategies for dermatology education: narrative review. JMIR Dermatol.

[25] Lehtoranta S, Xi N, Hamari J Gamification and employee well-being: a systematic literature review.

[26] Rash I, Shi K, Sussel R (2024). Virtual 3D simulation technology for interprofessional team training. Simul Gaming.

[27] Shi Y, Ferlet E, Crawfis R, Phillis P, Durano K 3D hospital: design and implement quest-based game framework for transitional training.

[28] RS 810.30 - loi fédérale du 30 septembre 2011 relative à la recherche sur l’être humain (loi relative à la recherche sur l’être humain, LRH). Fedlex.

[29] Commission cantonale d’éthique de la recherche sur l’être humain (CER-VD).

[30] Lockhart L (2020). Strategies to reduce nursing turnover. Nurs Made Incred Easy.

[31] Brook J, Aitken L, Webb R, MacLaren J, Salmon D (2019). Characteristics of successful interventions to reduce turnover and increase retention of early career nurses: A systematic review. Int J Nurs Stud.

[32] Kirk M (2017). Strategies for Health Care Administration Leaders to Reduce Hospital Employee Turnover.

[33] Németh J, Carron PN, Bentvelzen A, Gobron S Gamified-ED3D project.

[34] Lindner M, Leutritz T, Backhaus J, König S, Mühling T (2025). Knowledge gain and the impact of stress in a fully immersive virtual reality-based medical emergencies training with automated feedback: randomized controlled trial. J Med Internet Res.

[35] Survey of two apps to help new employee and reduce high turnover in health depts. Zenodo.

