# Supplementary figures and images for: Gamified Simulation for Onboarding Health Care Teams in Emergency Care: Development and Preliminary Feasibility Study
Source: JMIR Form Res. 2025 Dec 15;9:e72202. doi: 10.2196/72202 (PMC12704704; doi:10.2196/72202)

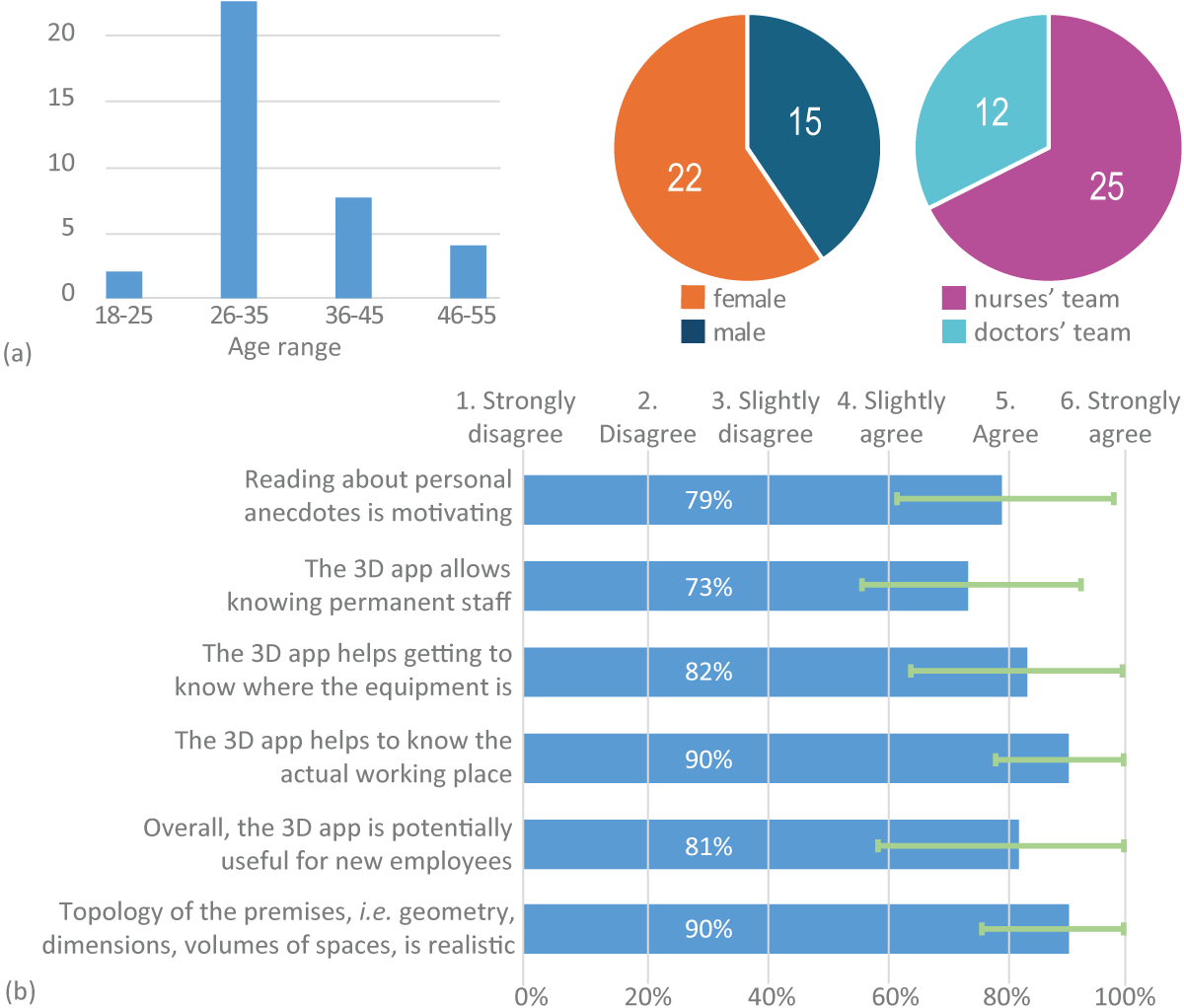

Supplement: Multimedia Appendix 1 [file formative-v9-e72202-s001.png]

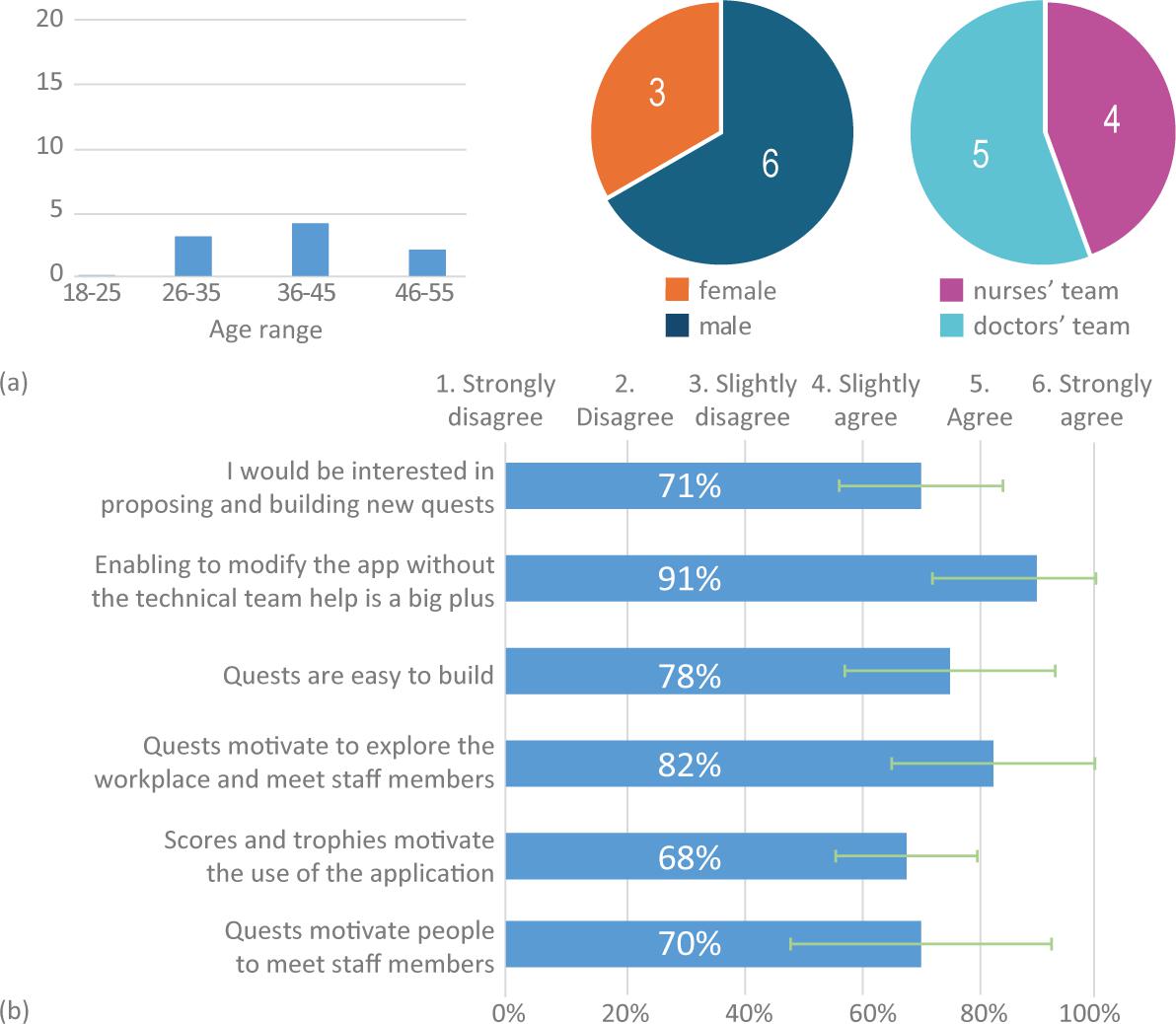

Supplement: Multimedia Appendix 2 [file formative-v9-e72202-s002.jpeg]
